# A pictorial series of interesting and unusual cases of breast malignancy encountered at triple assessment

**DOI:** 10.1186/1470-7330-15-S1-P19

**Published:** 2015-10-02

**Authors:** N Roszkowski, N Patel, R Oeppen, M Stahnke

**Affiliations:** 1 University Hospitals Southampton, Tremona Rd, Southampton, Hampshire SO16 6YD, UK

## Learning objectives

This pictorial series illustrates four cases with interesting imaging presentations of some unusual primary and secondary breast malignancies and aims to exemplify the range of appearances of malignancy and stimulate discussion of atypical differential diagnoses in appropriate cases.

## Content organisation

We present a pictorial series of four patients with unusual breast pathology who initially presented to the triple assessment breast clinic in a tertiary university institution. For each case the imaging features and final diagnoses are critically appraised, along with a review of the literature. We illustrate a case reminiscent of bilateral invasive ductal carcinoma which, after further histological immune profiling, is confirmed to be the first presentation of a metastatic lung cancer. A sarcomatoid carcinoma presents as a densely calcified well-defined lesion on mammography accompanying a synchronous invasive ductal carcinoma and could initially have been seen as benign. We present the imaging features of a fibroadenoma which interestingly was shown to contain DCIS. We also highlight the appearances of a recurrent pleomorphic invasive lobular carcinoma within latissimusdorsi muscle following mastectomy and reconstruction.

## Conclusion

Interesting and unusual differentials for malignant breast lesions are presented pictorially with an emphasis on the initial presentation and imaging findings. It is hoped that this will both educate and stimulate discussion of differential diagnoses in the breast imaging setting.

